# Supplementary material for: Biocontrol of Cercospora leaf spot in sugar beet by a novel Bacillus velezensis KT27 strain: Enhanced antifungal activity and growth promotion in laboratory and field conditions
Source: PLoS One. 2025 May 30;20(5):e0323889. doi: 10.1371/journal.pone.0323889 (PMC12124551; doi:10.1371/journal.pone.0323889)
Supplement: Attachment — Submitted filename: Manuscript Bacillus valezensis KT 27 induced by fungi.docx [file pone.0323889.s001.docx]

**Use of *Bacillus valezensis* KT 27 induced by fungi in the protection of sugar beets against Cercospora Leaf Spot (CLS)**

***Bacillus valezensis* KT 27 induced by fungi**

Agnieszka Wita^1^, Wojciech Białas^1^, Katarzyna Czaczyk^1^, Agnieszka Drożdżyńska^1^, Łukasz Sobiech^2^, Monika Grzanka^2^, Jakub Danielewicz^3^, Ewa Jajor^3^, Joanna Horoszkiewicz^3^, Roman Marecik^1^*

^1^ Department of Biotechnology and Food Microbiology, Poznan University of Life Sciences, Poland

^2^ Department of Agronomy, Poznań University of Life Sciences, Poznań, Poland

^3^ Department of Mycology, Institute of Plant Protection, National Research Institute, Poland

* Corresponding author

E-mail: roman.marecik@up.poznan.pl

**Abstract**

Fungal diseases in crops are a major cause of yield reduction and significant economic losses. Cercospora leaf spot (CLS) is one of the most serious diseases affecting sugar beet and other crops, caused by infection with the fungus *Cercospora beticola*. An increasing issue is the resistance of this pathogen to commonly used chemical fungicides, with elevated application rates significantly contributing to environmental pollution. In this study, the effect of the newly isolated strain *Bacillus valezenzis* KT27 on the growth of *Cercospora beticola*, *Rhizoctonia cerealis*, and *Fusarium oxysporum* was evaluated under laboratory conditions. The bacterium's ability to synthesize lipopeptides (surfactin, iturin, and fengycin), as well as its capacity to solubilization of phosphorus, potassium, and zinc, was also determined. In vitro tests demonstrated that *B. valezenzis* KT27 exhibited high efficacy in inhibiting *C. beticola* growth, with lower efficacy against *R. cerealis* and *F. oxysporum* (60.2%, 22.5%, and 15.5%, respectively). Centrifugation of bacterial biomass and the use of sterile supernatant in tests reduced the antagonistic effect by more than 3.5 times for all fungal species tested. A significant increase in the antagonistic activity of *B. valezenzis* KT27 against *C. beticola* was observed following the addition of thermally inactivated fungi (*C. beticola*, *R. cerealis*, and *F. oxysporum*) to the bacterial cultures. The biocontrol agents produced in this way were applied in field experiments to protect sugar beet against CLS. Results demonstrated a high efficacy of the bacterial preparation *B. valezenzis* KT27 induced by *R. cerealis*. In this case, the level of sugar beet protection was only 9.1% lower than that achieved using a combination of three different chemical fungicides. Furthermore, a positive effect of the *B. valezenzis* KT27 biopreparation on sugar beet growth and root yield was observed, with root yield up to 15.2% higher than in the untreated control group.

**Introduction**

Over the past decades, there has been a significant intensification of crop production to ensure food security for an increasing global population. Until now, this has largely been achieved through the use of successive generations of chemicals to protect plants against weeds, diseases, and pests. The widespread availability of pesticides and the use of mineral fertilizers have greatly increased the efficiency of plant production. However, these practices have progressively degraded the environment, leading to soil, air, and groundwater contamination, as well as eutrophication of water bodies. Furthermore, residues of these chemical compounds in plant tissues pose direct threats to the health of humans and animals [1, 2].

To address these issues, the European Commission introduced the 'European Green Deal,' which promotes non-chemical alternatives to improve soil quality and restore its biological balance while limiting the use of mineral fertilizers and chemical plant protection products. [3, 4]. The decision itself has led to a definite shift towards research undertaken as early as the mid-20th century, confirming the beneficial effects of selected microorganisms on the productivity of plant and animal production [4, 5]. The trend of replacing or supplementing chemical protection with biological agents is also visible in other regions of the world. This is reflected, among other things, in the growth in the value of the biopreparations market [6].

As a result, new solutions are currently being sought to replace agrotechnical practices that rely on chemical plant protection agents with more efficient and ecological alternatives that respect environmental quality and preserve ecosystem biodiversity. Biological methods of plant protection and the intensification of plant productivity are gaining increasing relevance in this context. These methods for controlling plant diseases and pathogens are mainly based on the diverse interactions of microorganisms, both direct and indirect. Antagonistic actions, direct competition for nutrients or other environmental elements, parasitism, and antibiosis are observed [7]. Selected microorganisms used as biocontrol agent produce substances such as the volatile organic compounds (VOCs), antibiotics, lipopeptides, enzymes, and phytohormones, which significantly enhance crop production efficiency [8-10]. Additionally, many microbial strains (microbial biocontrol agents) have the ability to fix atmospheric nitrogen or increase the availability of soil minerals, such as phosphorus, potassium, silica solubilization, and siderophore production [11].

Effective support in plan production strategies can be achieved by incorporating of specific “Palnt Growth Promoting Bacteria” (PGPB), such as those from the genus *Bacillus* [12, 13]. The genus Bacillus is a very large group of Gram-positive bacteria. They are found in various types of environments: they can be isolated from soil, fresh water, air, plant, or animal material. This is directly due to their exceptional adaptive abilities, allowing them to exist in aerobic or facultative anaerobic conditions, at high and low temperatures, high salinity, or low pH. The broad spectrum of metabolic activity of *Bacillus* bacteria has been exploited for the industrial production of many types of enzymes, medicinal/pharmaceutical compounds, and other active substances, including toxins [14]. The expansiveness and high metabolic activity of *Bacillus* bacteria cause that some of them can be dangerous — even to humans (e.g. *B. cereus* and *B. anthracis*). However, others, such as *B. amyloliquefaciens*, *B. subtilis*, *B. pumilus*, and *B. thuringiensis*, are used as biocontrol agents against other microorganisms that are plant pathogens [15, 16]. These strains have been successfully implemented as effective biocontrol agents in agricultural practice. Notably, in addition to their efficacy in inhibiting fungal, bacterial, and nematode growth, they exhibit the capacity for endospore formation. Endospores are highly resistant to adverse conditions such as UV radiation, extreme pH levels, temperature fluctuations or high salinity. This resistance of endospores is very important from a practical point of view because it significantly enhances the persistence of biocontrol agent in the environment, thereby prolonging their effectiveness [17].

One example of a strain with very high practical potential is *Bacillus velezensis*. *B. velezensis* (strain CR-502T and strain CR-14b) was first isolated from environmental samples collected at the mouth of the Vélez River in Torredelmar, in the province of Malaga, Spain [18]. Since then, reports have emerged of subsequent identifications of this strain in various environments, such as isolated from a wastewater sample collected from a fish farm, and from chicken cecal content [10, 19]. Phylogenetic analysis showed a high similarity of this strain to other species of the *Bacillus* genus, such as *B. subtilis* and *B. amyloliquefaciens* [19].

Genomic analysis of strain AMB-y1 of *B. velezensis* revealed that strains of this species produce metabolites with antibacterial, antifungal, and antibiotic activities, as well as tolerance to abiotic stress [20]. Genome analysis also showed that *B. velezensis* HNA3 is capable of synthesizing 14 secondary metabolites belonging to the groups: lipopeptides (fengycin, bacillomycin D, surfactin, mycosubtilin, and bacillibactin); polyketides (macrolactin, difficidin, and bacillaene); antibacterial peptides (bacilysin and amylocyclin); and other secondary metabolites including plipastatin, iturin, paenibactin, and paenilarvins [21, 22]. *B. velezensis* strain HNA3 has the ability to synthesize auxine - indole-3-acetic acid (IAA), which is nown as the hormone responsible for promoting plant growth [21]. *B. velezensis* T20E-257, isolated from tomato, produces 2,3-butanediol, which supports plant growth [23]. *B. velezensis* SQR9 colonizes plant roots and secretes metabolites that attract autochthonous microorganisms, contributing to biofilm formation [24].

These properties have made *B. velezensis* strains widely used as an alternative to traditional mineral fertilizers and as agents for controlling plant diseases caused by fungi or bacteria. A positive impact of preparations based on *B. velezensis* has been noted, for example, in the inhibition of wheat powdery mildew, *Fusarium* head blight (FHB), the biocontrol of *Phytophthora* diseases [16]. Currently, new strains of bacteria are being sought that could be used in agriculture. Work is also underway on solutions that will make them increasingly effective, including by creating an appropriate formulation that will allow for their proper application and uptake by plants. Another solution is to induce bacteria with fungi, which can potentially increase the antifungal activity of microorganisms used in agriculture [25, 26].

One of the most serious diseases of sugar beet (*Beta vulgaris* sp. *vulgaris*) and other species of the *Beta* genus, causing immense damage worldwide, is leaf spot caused by the fungal pathogen *Cercospora beticola* (CLS - Cercospora Leaf Spot). In addition, susceptibility to this disease is also observed in species from the families: *Chenopodiaceae*, *Acanthaceae*, *Apiaceae*, *Asteraceae*, *Brassicaceae*, *Malvaceae*, *Plumbaginaceae*, and *Polygonaceae* [27]. Initially, older leaves are affected, and as the disease progresses, younger leaves become infected as well. Leaves infected by *C. beticola* develop circular, ring-shaped discolorations. Depending on the stage of the disease, the discolorations can range in color from light brown or gray to red-purple and yellow. Due to the metabolic activity of the fungus, necrotic changes occur within the discolorations, leading to a reduction in the photosynthetic area, wilting, and eventually the death of infected leaves. Infected fields experience a significant reduction in yield (up to 40%), and the harvested raw material is of lower technological value for sucrose extraction [28]. The occurrence of the disease is associated with large economic losses worldwide [29, 30]. For this reason, combating CLS is very important, especially since the complete life cycle of *C. beticola* leads to the production of forms such as pseudostromata or false stromata (conidia-producing hyphae), which remain active in the soil and capable of infecting plants for up to two years. In practice, to avoid infection, it is recommended to use appropriate crop rotation, including the use of plants resistant to *C. beticola*. However, most commonly, after the first symptoms of the disease are observed, chemical protection is implemented using appropriate fungicides. Unfortunately, despite the temporary effectiveness of chemical treatments, there is an observed phenomenon of the pathogen resistance to the active ingredients in the fungicides, necessitating increased doses or more often, a change in the active substance [31, 32].

The presented study describes the characteristics of the newly isolated strain of *B. velezensis* KT 27 in the context of its suitability for producing a biocontrol agents for sugar beet protection against the *Cercospora beticola* infection and CLS disease. The effectiveness of *B. velezensis* KT 27 was tested with and without induction by inactivated cultures of *Cercospora beticola*, *Rhizoctonia cerealis*, and *Fusarium oxysporum*. Additionally, the antagonistic effect of B. valezensis KT27 on the growth of *Rhizoctonia cerealis* and *Fusarium oxysporum* was also determined.

**Materials and Methods**

*Microorganisms strains*

In the experiments, the *Bacillus velezensis* strain KT 27, isolated from compost derived from the fermentation and composting of selectively collected biodegradable waste—including green waste from the maintenance of green spaces, gardens, and parks, as well as kitchen waste—was utilized. Initially, the isolated strain was identified based on its morphological characteristics, and then confirmed using the MALDI-TOF MS (Matrix-assisted laser desorption/ionization time-of-flight mass spectrometry) technique, and further validated by analysis of a fragment of the 16S rDNA. Fungal isolates *(Cercospora beticola*, *Fusarium oxysporum*, *Rhizoctonia cerealis*) were obtained from the National Research Institute - Institute of Plant Protection in Poznań, Poland.

*Cultivation of miocroorganisms*

The *B. valezensis* KT27 bacteria strain was cultured in tryptone soya broth (TSB, Becton, Dickinson and Co., Franklin Lakes, NJ, USA)) medium for 48 hours at 37°C on a rotary shaker (250 rpm). The number of bacteria cells was analysed by pour plate method using Tryptone Soya Agar medium (TSA, Becton, Dickinson and Co., Franklin Lakes, NJ, USA) incubated at 30°C for 48h. After incubation every single colony was counted and the results was expressed as colony forming units (CFU). *F. oxysporum*, *R. cerealis* and *C. beticola* were cultivated on yeast extract peptone dextrose (YPD) medium for 5 days at 26°C on a rotary shaker (180 rpm).

*B. valezenzis KT27 antifungal efficiacy analysis*

The *B. valezenzis* KT27 strain was evaluated for their antifungal activity against *C. beticola., F. oxysporum*, *R. cerealis* and, which are one of the major pathogens causing crop yield losses. After 48h of bacteria cultivation and reaching a cells concentration of 1.0E+08 CFU/ml, one part of the bacterial suspension was collected the second was centrifuged at 4500 rpm for 10 minutes and then supernatant was filtered using syringe filters with a pore size of 0.45 µm (Merck, Darmstadt, Germany). The antagonistic activity of *B. valezenzis* KT27 against *C. beticola, F. oxysporum* and *R. cerealis* fungi was assessed using the well method. Petri dishes were filled with 20 cm³ of potato dextrose agar medium (PDA, Oxoid, Milan, Italy) at 55°C inoculated with 2% of the tested fungi inoculum. After solidification, two wells of 10 mm diameter were cut into the medium. One well was inoculated with 100 µl of a 48-hour bacterial culture, while the other with 100 µl of sterile supernatant obtained by centrifugation and filtration. Plates were then incubated at 26°C. After 4 days of incubation, the diameters of the fungi growth inhibition zones around the wells were measured. The level of fungi growth inhibition was assessed in millimetres and expressed as a percentage relative to the control sample (walls inoculated by 100 ml of sterile deionised water). Representative examples of antagonistic effects are illustrated in Figure 1.

Figure 1. Samples of antagonistic activity of *B. valezenzis* KT27 against (a) *C. beticola*, (b) *R. cereralis* and (3) *F. oxysporum*. On each photo left well represents cell suspension activity, right - supernatant activity.

*Enhancement of B. valezenzis KT27 activity against C. beticola*

As the effect of separate study (data unpublished) the TSB medium for *B. valezenzis* KT27 was enriched with 20 g/l of mannitol and 5 g/l of yeast extract. Additionally, to induce the metabolic activity of the bacteria, the medium was supplemented by 10% of volume the 5 days culture of *C. beticola., F. oxysporum* or *R. cerealis* inactivated by autoclaving at 121°C for 30 min. Such prepared medium was inoculated by *B. valezenzis* KT27 (5% of total medium volume, cells concentration 1.0E+08 CFU/ml) and cultivation was carried out for 48 h at 37°C on a rotary shaker (250 rpm). Obtained cultures were used to analysis of bacterial antagonistic activity against *C. beticola* strain.

*Quantitative analysis of phosphate solubilization*

*B. velezensis* KT 27 strain was subjected to quantitative determination of phosphate solubilization. In the first step 48 hours *B. valezensis* KT27 bacterial suspension was centrifuged (4500 rpm; 10 min) to avoid skewing the results by dissolved phosphates presents in the culture medium. Then, the supernatant was decanted, and the biomass was suspended by use of 6 ml of water. The next step was to prepare the 100 ml liquid Pikovskaya (PVK) medium containing 2,5 g/l tricalcium phosphate (TCP) as insoluble inorganic form of phosphorus [33] and inoculating with 2 ml of the previously prepared inoculum. The cultures were shaken for 14 days at 30°C. Samples were taken under aseptic conditions and centrifuged for 15 minutes at 4500 rpm to remove sediments. A soluble phospohorus concentration in the samples was analysed using SPECTROQUANT Phosphate Cell Test (cat. no.: 1.00673.0001, within the range: 9-307 mg/L PO₄³⁻, Merck, Darmstadt, Germany). To the kit reaction vial, 0.20 ml of the analysed sample, 1 dose of P-1K reagent were added, and mixed. The vial was then heated at 120°C for 30 minutes in a thermoreactor (Spectroquant TR 420, Merck, Darmstadt, Germany). After cooling, 5 drops of P-2K were added, mixed, and then 1 dose of P-3K was added, mixed again, and left for 5 minutes. Finally, PO₄³⁻ levels in the samples were measured using a spectrophotometer (Spectroquant Pharo 100, Merck, Darmstadt, Germany).

*Quantitative assessment of potassium solubilization*

*B. velenzensis* KT 27 strain was examined for their potassium solubilizing activity. The inoculum prepared as for phosphorus analysis was used for inoculation of Aleksandrov's broth medium containing 2 g/l Muscovite (KAl₂(AlSi₃O₁₀)(OH)₂) as e source insoluble potassium according to Rajawat et al. [34]. The culture was carried out for 14 days at 30°C. During the culture samples were collected, centrifuged and supernatants were used for the released potassium concentration analysis. The ability of the B. velenzensis KT 27 to solubilize potassium was determined using the SPECTROQUANT Potassium Cell Test (cat. no.: 1.14562.0001, range: 5.0-50.0 mg/l K, Merck, Darmstadt, Germany). The turbidimetric method was based on the photometric measurement of the soluble precipitate formed in the reaction of potassium ions in an alkaline solution react with sodium tetraphenylborate. Analysis were performed strict to producers manual.

*Quantitative assessment of zinc solubilization*

The ability of the *B. velenzensis* KT 27 to solubilize zinc was determined using the SPECTROQUANT Zinc Cell Test (cat. no.: 1.14566.0001, range: 0.20-5.00 mg/l Zn, Merck, Darmstadt, Germany). The method was based on the photometric measurement of the colour reaction between zinc ions and pyridylazo-resorcinol (PAR) in an alkaline solution. Bacteria for the test was cultured on Bunt and Rovier broth medium containing 1 g/l of ZnO [35]. During 14 days of the culture at 30°C samples were collected, centrifuged and supernatants were used for the released zinc concentration analysis. Analysis were performed strict to producers manual.

Bioreactor cultivation and biopreparation of *B. valezenzis* KT27 production

The biomass production was carried out in 5-liter bioreactors (Biostat B Plus, Sartorius Stedim Biotech GmbH, Germany). The bioreactors, with the modified TSB medium (additional carbon/nitrogen source in the form of 20 g/l of mannitol and 5 g/l of yeast extract) in a volume of 2 l, were sterilized in an autoclave at 121°C for 30 minutes. After installation at the workstation and cooling to 30°C, the medium was inoculated by the *B. valezenzis* KT27 cells, which constituted 5% of the medium's volume. The bacterial culture in the bioreactors was conducted at 30°C for 72 hours. During cultivation, the pH was maintained at 7 by automatic dosing system of 0.1% HCl or 0.1% NaOH. Saturation at a level of 30% dissolved oxygen was achieved by constant aeration at a rate of 1 vvm (vessel volumes per minute) and mixing at a range of 100 to 500 rpm. Under analogous conditions, cultures of *B. valezenzis* KT27 were also carried out with simultaneous induction of antifungal activity. For this purpose, immediately after introducing the bacterial inoculum, 200 ml of inactivated (sterilized in an autoclave at 121°C for 30 minutes) culture of *C. beticola*, *F. oxysporum* or *R. cerealis* was added to the medium. During cultivation, samples were taken to analyze fungistatic activity and the concentration of metabolites responsible for inhibiting pathogen development. To obtain a biocontrol agents containing *B. valezenzis* KT 27 for field experiments, a culture was also conducted under analogous conditions in a 30-liter bioreactor (Biostat C Plus, Sartorius Stedim Biotech GmbH, Germany).

*Determination of surfactin, iturin, fengycin in culture of B. velezensis KT 27*

To determine surfactin, iturin and fengycin in culture fluids, the 50 ml of the 48 h culture was centrifuged at 4600 RPM for 20 minutes, and the supernatant was transferred to a new 50 ml Falcon tube. The supernatant volume was precisely measured and adjusted to a final concentration of 25% methanol (Merck, Darmstadt, Germany). Next, the sample was purified using SPE (Solid Phase Extraction) columns (Agilent Technologies, Inc., Santa Clara, CA, USA). The obtained eluate was evaporated, and the residue was dissolved in 1 ml of methanol. Finally, before chromatographic analysis, the sample was filtered using 0.22 µm pore size nylon syringe filters (Merck, Darmstadt, Germany). Chromatographic analysis of surfactin, iturin, and fengycin was performed on an Agilent Technologies 1200 Series A HPLC (Agilent Technologies, Santa Clara, CA, USA). Lichrosphere 100 RP18 250-4 column (Merck, Darmstadt, Germany) was used, and the column was eluted with a gradient of (A) 0,76mM trifluoroacetic acid (TFA) (Sigma-Aldrich, St. Louis, MO, USA) in water and (B) 0,76mM TFA in acetonitrile (Merck, Darmstadt, Germany) at a flow rate as follows: 0 min – 45% B; 3 min – 50% B; 8 min – 80% B; 25 min – 100% B; 30 min – 100% B; 31 min – 45% B. The mobile phase flow rate was 1 ml/min. The analysis was conducted at 30°C with a wavelength of 210 nm. Qualitative and quantitative analyses were performed using an external standard method with the ChemStation for LC 3D Systems software from Agilent Technologies (Agilent Technologies, Inc., Santa Clara, CA, USA).

Table 1. Meteorological conditions during the research

| **Month-year** | **Weather parameters** | **Decade** | | | **Average/total** |
| --- | --- | --- | --- | --- | --- |
|  |  | I | II | III |  |
| 03-2023 | Average temperature [^o^C] | 0,74 | 5,88 | 8,08 | **4,90** |
|  | Average air humidity [%] | 91,16 | 77,83 | 79,60 | **82,86** |
|  | Total rainfall [mm] | 8,40 | 0,50 | 0,00 | **8,90** |
| 04-2023 | Average temperature [^o^C] | 4,85 | 9,50 | 10,42 | **8,21** |
|  | Average air humidity [%] | 81,35 | 85,55 | 72,83 | **79,83** |
|  | Total rainfall [mm] | 0,30 | 0,00 | 0,00 | **0,30** |
| 05-2023 | Average temperature [^o^C] | 10,20 | 13,71 | 16,13 | **13,35** |
|  | Average air humidity [%] | 66,12 | 75,35 | 63,11 | **68,19** |
|  | Total rainfall [mm] | 12,00 | 16,30 | 0,10 | **28,40** |
| 06-2023 | Average temperature [^o^C] | 18,40 | 18,71 | 20,08 | **19,06** |
|  | Average air humidity [%] | 53,47 | 66,15 | 74,79 | **64,80** |
|  | Total rainfall [mm] | 0,20 | 6,60 | 36,30 | **43,10** |
| 07-2023 | Average temperature [^o^C] | 20,66 | 21,65 | 18,83 | **20,38** |
|  | Average air humidity [%] | 61,06 | 62,34 | 77,30 | **66,90** |
|  | Total rainfall [mm] | 1,70 | 10,20 | 26,90 | **38,80** |
| 08-2023 | Average temperature [^o^C] | 17,51 | 23,58 | 19,28 | **20,12** |
|  | Average air humidity [%] | 78,89 | 69,78 | 81,38 | **76,68** |
|  | Total rainfall [mm] | 36,50 | 2,70 | 25,80 | **65,00** |
| 09-2023 | Average temperature [^o^C] | 19,10 | 19,24 | 17,65 | **18,66** |
|  | Average air humidity [%] | 72,96 | 73,11 | 75,40 | **73,82** |
|  | Total rainfall [mm] | 0,00 | 6,20 | 5,70 | **11,90** |
| 10-2023 | Average temperature [^o^C] | 13,01 | 9,92 | 11,05 | **11,33** |
|  | Average air humidity [%] | 66,10 | 83,06 | 92,83 | **80,67** |
|  | Total rainfall [mm] | 11,00 | 8,10 | 17,50 | **36,60** |

*Controlling of sugar beet CLS disease by biological agent based on B. valezenzis KT27– the field study*

The field experiment was conducted in Winna Góra (Wielkopolskie Province, Poland). The meteorological conditions prevailing during the development of sugar beets are presented in Table 1.

The soil of the test field was classified as soil quality class IIIb, with a pH of 6.0 and an organic matter content of 1.67%. The experiment was carried out in a randomized block design with four replications for each combination. The plots measured 3 m x 10 m = 30 m². Conventional farming practices were followed on the experimental field. The sugar beet variety Kujavia was sown on April 6, 2023, with a row spacing of 45 cm. Mineral fertilization was planned based on soil fertility and the nutritional requirements of the plants (Table 2). The experiment was designed and conducted in accordance with EPPO guidelines: PP 1/1(4), PP 1/135(4), PP 1/152(4), PP 1/181(5), PP 1/225(2) [36-40].

Table 2. Fertilization timing and dosages in sugar beet cultivation

| **Application dates** | **Nutrients concentration (kg x ha^-1^)** | | | |
| --- | --- | --- | --- | --- |
|  | **N** | **P** | **K** | **S** |
| 28.03.2023 | 50,6 |  |  |  |
| 28.03.2023 | 15 | 50 | 75 | 17,5 |
| 28.03.2023 |  |  | 140 |  |
| 28.03.2023 |  | 45 |  |  |
| 12.05.2023 | 54,4 |  |  |  |

Treatments aimed at controlling sugar beet CLS disease were performed three times. The fungicides used in the comparative combination were Dafne 250 EC (difenoconazole - 250 g/l, INNVIGO Sp. z o.o., Poland), Makler 250 SE (azoxystrobin - 250 g/l, INNVIGO Sp. z o.o., Poland), and Tebu 250 EW (tebuconazole - 250 g/l, Helm AG, Germany). The test of biological agent contained strain *B. valezenzis* KT27 or *B. valezenzis* KT27 obtained in culture under induction conditions (additive of inactivated *C. beticola*, *F. oxysporum* or *R. cerealis*). The timing of the treatments and the doses of the applied products are provided in Table 3. The treatments were carried out using a backpack sprayer equipped with TeeJet 110 03 VXR nozzles. The spray liquid output was 200 l/ha, the boom height above the plants was 50 cm, and the working speed was 5 km/h. Herbicides and insecticides were applied according to current recommendations.

The effectiveness of biological agent was assessed by estimating the average percentage of affected leaf area on 25 randomly selected sugar beet plants in each plot. The effects of the applied biocontrol agent were evaluated visually by comparing the condition of plants in treated plots with those in control plots. The intensity and extent of plant damage under biological or chemical protection treatments were evaluated relative to an untreated control sample, with results expressed as percentages. Root yield was harvested on October 10, 2023, and then weighed on a technical scale.

Table 3. Timing and dosages of chemical fungicides and biological treatments in sugar beet cultivation

| No. | Fungicide/ biocontrol agents | Application data  (T1, T2, T3) | Dose of  fungicide/  biocontrol agents  (L x ha^-1^) |
| --- | --- | --- | --- |
| 1 | Control (no protection) | - | - |
| 2 | T1 - difenoconazole  T2 - azoxystrobin  T3 - tebuconazole | T1-27.07.2023  T2-17.08.2023  T3-02.09.2023 | T1 - 0.4  T2 - 1.0  T3 - 0.8 |
| 3 | T1, T2, T3 – *B. valezensis* KT27 | T1-27.07.23  T2-10.08.23  T3-25.08.23 | T1, T2, T3 - 0.5 |
| 4 | T1, T2, T3 - *B. valezensis* KT27 + *C. beticola* |  | T1, T2, T3 - 0.5 |
| 5 | T1, T2, T3 - *B. valezensis* KT27 + *R. cerealis* |  | T1, T2, T3 - 0.5 |
| 6 | T1, T2, T3 - *B. valezensis* KT27 + *F. oxysporum* |  | T1, T2, T3 - 0.5 |

After conversion, the result was expressed in tons per hectare. Statistical analysis of the data was conducted using ANOVA to assess differences among treatment groups. Post-hoc comparisons were performed using Tukey's Honest Significant Difference (HSD) test to identify specific pairwise differences. Significance levels were set at p ≤ 0.05, and all analyses were performed using OriginPro 2024b statistical software (USA). Results are reported as mean ± standard deviation, with significant differences among groups indicated in the figures. Effectiveness was calculated according to Abbott's formula.

**Results**

*Antagonistic activity*

The results of laboratory tests showed that the bacterial strain *Bacillus valezensis* KT27 exhibits antagonistic properties against the pathogenic *Cercospora beticola*, which causes the dangerous CLS disease in sugar beet crops and other plants in the *Asteraceae* family. This confirms the exceptional potential of *Bacillus* bacteria, making these microorganisms suitable for use as components of biocontrol agent applied in plant protection agro-technology [41]. Using the well diffusion method for testing the antagonistic activity of microorganisms, it was found that the presence of *B. valezensis* KT27 cells strongly inhibited the growth of *C. beticola* around the well (Fig. 2). The strongest inhibition effect, at a level of 60,20%, was observed with the liquid from a 48-hour culture of *B. valezensis* KT27, while separating the cells by centrifugation and filtration (ø 0.22 µm) reduced the effectiveness of *C. beticola* growth inhibition by about 69,25% (Fig. 1). Much weaker antagonistic inhibition effects of *B. valezensis* KT27 on growth other mold fungi; *F. oxysporum* and *R. cerealis* was observed. In this cases the antagonistic effect was 3,9 and 2,7 times weaker than against *C. beticola*, respectively. Additionally, it was observed that sterile supernatants from the *B. valezensis* KT27 cultures showed more then 3,5 times lower inhibition of tested fungi growth.

Figure 2. Antagonistic activity of *Bacillus valezenzis* KT27 against *Cercospora beticola*. The graph displays the inhibitory effects on *C. beticola* in terms of biomass (cell suspension) and supernatant fractions. Statistical significance is denoted as follows: *p* ≤ 0.05 (*)*, p ≤ 0.01* (**)*, and* *p* *≤ 0.001* (***)

Studies were also conducted to assess the impact of the tested fungal strains on enhancing the antagonistic properties of *B. valezenzis* KT27. For this purpose heat-inactivated culture of *C. beticola*, *F. oxysporum* or *R. cerealis* were added to the culture of *B. valezenzis* KT27. The results confirmed the positive effect of nearly all inactivated fungi on the activity of *B. valezenzis* KT27 (Fig. 3). A lack of bacterial sensitivity was observed only with the addition of *F. oxysporum* to the medium. Surprisingly, the highest increase in the activity of *B. valezenzis* KT27 against *C. beticola* was not observed when induced by inactivated *C. beticola* itself, but rather by the presence of inactivated *R. cerealis* in the bacterial culture. The addition of *R. cerealis* to the *B. valezenzis* KT27 culture resulted in a 33.48% increase in the inhibition of *C. beticola* growth, whereas the introduction of inactivated *C. beticola* led to an increase of only 13.75% (in relation to control - no induced bacteria culture). Similar changes in antifungal activity were observed in sterile supernatants obtained by centrifugation and filtration of the *B. valezenzis* KT27 culture. In this case the greatest induction of antagonistic activity was also observed in samples obtained after culturing the bacteria with the addition of inactivated *R. cerealis* culture.

Figure 3. Antagonistic activity of *Bacillus valezenzis* KT27 against *Cercospora beticola*, with and without induction by inactivated cultures of *C. beticola*, *Rhizoctonia cerealis*, and *Fusarium oxysporum*. The graph displays the inhibitory effects on *C. beticola* in terms of biomass (cell suspension) and supernatant fractions. Statistical significance is denoted as follows: *p* ≤ 0.05 (*)*, p ≤ 0.01* (**)*, and* *p* *≤ 0.001* (***)

*The effect of B. valezenzis KT27 on phosphorus, potassium and zinc solubiliozation*

Cultivating *B. valezenzis* KT27 for 14 days on special media containing insoluble forms of phosphorus, potassium, and zinc confirmed the ability of the studied strain to convert these compounds into water-soluble forms, which are a potential source of these elements and essential for high plant yield (Fig. 4). The amount of released phosphorus and potassium in particular was strongly correlated with bacterial growth (OD600). In the initial hours of cultivation, the concentration of phosphorus and potassium ranged from 5.41 to 10.45 mg/l and 5.25 to 15.74 mg/l, respectively. As bacterial growth entered the logarithmic phase, the concentration of these elements rapidly increased reaching a maximum of 53.26 mg/l for phosphorus and 45.83 mg/l for potassium at 36 hours of cultivation, however potassium being released faster than phosphorus. In the stationary phase, where OD600 remained constant, no further increase in the solubilization of phosphorus and potassium was observed. A significantly lower level of solubilization for zinc was noted. Depending on the cultivation time, the *B. valezenzis* KT27 strain released only between 0.22 and 5.99 mg/l of this element.

Figure 4. Solubilization of phosphorus, potassium, and zinc by *Bacillus valezenzis* KT27 in relation to bacterial growth kinetics. The graph illustrates the solubilization levels of each mineral (mg/L) over time alongside the growth curve of *B. valezenzis* KT27, measured as optical density at 600 nm.

*Determnation of surfactin, iturin, and fengycin in culture B. velezensis KT 27*

The chromatographic analysis of the culture fluids from *B. valezenzis* KT27 bacteria and bacteria with the addition of inactivated fungi from the species *C. beticola*, *F. oxysporum*, or *R. cerealis* revealed the presence of lipopeptides such as surfactin, iturin, and fengycin. Overall, the concentration of these compounds was low (Fig. 5). The highest concentrations were recorded for surfactin, ranging from 10.2 to 12.8 mg/l, depending on the type of culture. In the case of iturin and fengycin, significantly lower amounts were detected, with concentrations not exceeding 1.99 mg/l.

Figure 5. Production levels of key lipopeptides in cultures of *Bacillus valezenzis* KT27 strain, both with and without induction by inactivated cultures of *Cercospora beticola*, *Rhizoctonia cerealis*, and *Fusarium oxysporum*.

*CLS control in the sugar beet culture - field study*

A very important aspect of the research was confirming the effectiveness of a biocontrol agent composed of *B. valezenzis* KT27 bacterial strain cells in protecting sugar beets against CLS disease caused by *C. beticola* under field conditions. For this purpose, 72 hours of bioreactor cultures of the pure bacterial strain and bacteria induced by inactivated fungi from the species *C. beticola*, *F. oxysporum* or *R. cerealis* were conducted, resulting in a bioactive agents used in sugar beet protection. By enriching the TSB medium with 20 g/l of mannitol and 5 g/l of yeast extract, a higher concentration of microorganisms (1.00E+09 CFU) was obtained. The biocontrol agents were applied directly to infected plants, and the effects were observed. The results were compared to a control sample without any protection and to plants treated with chemical control of CLS disease (difenoconazole, azoxystrobin, tebuconazole).

Field studies provided results confirming the positive impact of *B. valezenzis* KT27 in reducing the effects of *C. beticola* infection and, consequently, limiting the spread of CLS disease (Fig. 6). As in laboratory studies, the most effective variant against *C. beticola* was the one where antifungal activity was induced by adding an inactivated suspension of *R. cerealis* to the bacterial culture. The use of this biocontrol agent (0.5 l/ha) resulted in up to 96.4% effectiveness in controlling CLS in the first term (T1) compared to the control (without protection). It is noteworthy that the biocontrol agents effectiveness at this time was 11% higher than chemical protection in the form of difenoconazole. A high level of control against *C. beticola* (52.9% and 51.5%) was maintained in subsequent applications dates (T2 and T3). Ultimately, the effectiveness of the biocontrol agent was only 9.1% lower than the three different chemical active substances (difenoconazole - 250 g/l, azoxystrobin - 250 g/l, tebuconazole - 250 g/l) applied successively in subsequent terms. Significantly lower effectiveness against *C. beticola* was observed when using biopreparations based on *B. valezenzis* KT27 and bacteria induced by the addition of inactivated *C. beticola* or *F. oxysporum*. After the last application (T3), the non-induced *B. valezenzis* KT27 agent resulted in only a 12.1% reduction in CLS occurrence compared to the control, and this effectiveness was 50% lower than that of chemical preparations. It is important to note that, regardless of the type of biopreparation used, its highest effectiveness was observed after the first application to the youngest plants, ranging from 26.4% for non-induced *B. valezenzis* KT27 to 96.4% for the *B. valezenzis* KT27 suspension induced by *R. cerealis*.

Figure 6. Biocontrolling of CLS disease using different treatment variants of *Bacillus velezensis* KT27 culture compared to standard chemical protection. T1, T2, and T3 indicate application dates.

The high level of protection of sugar beet crops against CLS was directly reflected in the yield of the roots. Regardless of the type of control agent used, the sugar beet root yield was significantly higher than in unprotected crops. Despite marked differences in CLS control effectiveness among the various biocontrol formulas, a comparable root yield of 67.31 – 72.99 t/ha was achieved, which was 6.5 - 15.2% higher than the control sample. Nonetheless, the highest root mass (72.99 t/ha) was harvested in the beet crops treated with the formula containing *B. valezenzis* KT27 bacteria induced by *R. cerealis*. This result was only 10.8% lower than in crops protected chemically (81.72 t/ha).

**Discussion**

Fungal diseases lead to a reduction in the productive capacity of plants, contribute to the deterioration of the quality of harvested products, and in some cases, result in their complete disqualification as food products. On a global scale, this causes massive economic losses, and locally, they may threaten the existence of people and livestock. One, such serious threat is CLS disease caused by the infection of the fungus *C. beticola*. Currently, one of the most promising ways to mitigate the effects of fungal diseases in plant cultivation is the use of biological methods, and a key element of this approach is the use of bacteria, including those from the *Bacillus* genus [12, 13, 15, 16, 42]. In the presented study, we analyzed the potential use of the *B. valezenzis* KT27 strain as an ecological solution for protecting sugar beet against *C. beticola* infection and its effects. Some bacterial properties responsible for the plant growth promotion effect were also examined. In laboratory tests, we confirmed both the bacteria’s ability to solubilize essential plant nutrients and its strong antagonistic effect on the growth of *C. beticola*. The laboratory results were confirmed under real sugar beet cultivation conditions.

Previous research indicates that various factors significantly influence the antagonistic interactions of microorganisms [4, 8]. In our study, we demonstrated that the natural antagonistic activity of *B. valezenzis* KT27 against *C. beticola* could be enhanced by adding thermally inactivated fungal cultures to the bacterial cultures. As a result of such an action (addition of inactivated *R. ceraelis*), more than a 50% increase in the bacteria's antifungal activity was observed. This type of reaction is supported by literature data. In the research by de Boer et al. [43], it was shown that the fungus *Mucor hiemalis* positively influenced the growth of *Achromobacter* and *Stenotrophomonas* bacteria, which exhibited strong antagonism towards the plant-pathogenic fungus *Rhizoctonia solani*. The authors of the publication advocate for the introduction of *M. hiemalis* into the soils of cultivated plants as a means of biocontrol against soil-borne pathogenic fungi [43]. It is suspected that this effect may be due to increased activity of chitinolytic enzymes involved in the destabilization and degradation of chitin, a key component of fungal cell walls [44]. This type of induction of bacterial antifungal activity was also observed in cases where spores of *Plasmodiophora brassicae* were introduced into the soil [45].

In our case, metabolic activity of *B. valezenzis* KT27 associated with the production of substances, including those with antifungal activity, was also noted. Chromatographic analysis confirmed the presence of cyclic lipopeptides (CLPs) such as iturin, fengycin, and most notably surfactin. The ability of *Bacillus* species to secrete such compounds is supported by numerous publications [46-48]. Lipopeptides produced by bacteria can disrupt the integrity of cytoplasmic membranes, leading to a disruption of the phytopathogen's cell membrane, potentially causing cytoplasmic leakage, hyphal death, or inhibition of spore germination [49].

In the presented studies, surfactin was the lipopeptide with the highest concentration in all experimental variants. However, its highest recorded concentration was only 18,8 mg/l, while literature reports confirm production levels of this compound reaching several grams per liter [50]. Although the antibacterial and antifungal properties of surfactin are not as strongly confirmed in the literature as those of iturin or fengycin, its presence triggers both specific and non-specific cellular defense mechanisms. Research by Lilge et al. [51] demonstrated that the impact of high concentrations of surfactin (100 g/l) on the bacterial cell wall induces protein expression comparable to the response observed with antibiotics such as bacitracin, nisin, ramoplanin, vancomycin and daptomycin. The inhibition of *Fusarium foetens* mycelial growth by 50.2% and 45.6%, respectively, in *in vitro* and *in vivo* studies under the influence of surfactin (20 g/l) from *Bacillus subtilis* SF1 was also reported by Liu et al. [52]. In this case, changes in protein expression and an increase in reduced glutathione production were also observed. Interestingly, even heating bacterial cultures to 100°C did not significantly reduce antifungal activity, indicating a different mechanism of action than one linked to the hydrolytic activity of enzymes. Although surfactin itself does not directly damage the pathogen's DNA like fengycin or iturin, it undoubtedly plays a protective role for other lipopeptides and enhances their effectiveness in penetrating the cell [53].

Even very high efficacy of a biopreparation under in vitro conditions must be confirmed in real-world conditions (field studies). Our in vivo studies on the protection of sugar beet crops from CLS using a biocontrol agents spray consisting of a suspension of *B. valezenzis* KT27 cells yielded promising results. In the best experimental variant (*B. valezenzis* KT27 + *R. cerealis*), approximately 50% inhibition of CLS development was observed compared to the control, with only 9.1% lower efficacy than the combination of three different chemical active substances (difenoconazole, azoxystrobin, tebuconazole). Additionally, up to a 15% increase in sugar beet root yield was noted compared to the control, which is undoubtedly due to the reduced activity of *C. beticola*. However, it is also attributed to the ability of *B. valezenzis* KT27 to solubilize phosphorus and potassium, thereby enhancing the availability of these elements to the plant. These results are consistent with other studies on the use of *Bacillus* species against plant fungal diseases.

El Housni et al. [54] conducted screening of microorganisms for their antagonistic effect on *C. beticola* activity. Among the 18 isolated strains, as many as 50% exhibited antifungal properties, and 6 of them inhibited fungal growth by up to 80%. 16S RNA sequencing enabled the identification of the microorganisms and assigned 4 of them to the *Bacillus* genus. Genetic and biochemical analysis of the *Bacillus* strains revealed the presence of genes encoding sequences responsible for the production of antifungal metabolites: bacillomycin, iturin, fengycin, and surfactin. Similar to the results obtained by our team, El Housni et al. [55] demonstrated a positive effect of a *Bacillus*-based preparation on sugar beet growth under field conditions. An increase in root length, stem length, and overall plant biomass was observed, along with a 77.42% efficacy in controlling CLS, which was only slightly lower than the protection provided by the chemical formula difenoconazole (88.51%).

The high efficacy of the *Bacillus* strain RB2 in controlling *C. beticola* (60.86%) in sugar beet cultivation was also demonstrated by Arzanlou et al. [56]. Additionally, in greenhouse experiments, they showed that the preventive use of a formula containing *Bacillus* RB2 protected sugar beet plants from *C. beticola* infection and the effects of CLS disease by 96% [55]. Collins et al. [17] research suggests that many factors may influence the effectiveness of sugar beet protection against CLS. Using vegetative cells significantly increased the plants resistance to *Cercospora* infection compared to the use of *B. subtilis* spores [17]. The impact of 17 strains of *B. amyloliquefaciens* isolated from the sugar beet phyllosphere against *C. beticola* was tested by Esh et al. [56]. They reported a positive effect of these bacteria in inhibiting CLS disease, particularly when used preventively—before the fungal pathogen appeared. Soliman et al. [57], in both in vitro and in vivo studies, demonstrated approximately 40% fungicidal efficacy of *B. amyloliquefaciens* RaSh1 against pathogenic *A. alternata*, which, like *C. beticola*, causes leaf wilting. On the other hand, studies on the use of *Moringa oleifera* extract, potassium bicarbonate, and *B. subtilis* in combating *C. beticola* showed that *B. subtilis* was the least effective method for protecting sugar beets from the development of CLS [58].

**Conclusion**

The protection of cultivated plants from diseases caused by various fungal infections is essential to ensure adequate production levels and profitability. However, the extensive use of chemical plant protection products negatively affects the environment, biodiversity, and often the quality and safety of the produced food. For these reasons, there is growing interest in exploring alternative, biological methods to eliminate the causes and consequences of plant diseases. *Bacillus* bacteria undoubtedly have the potential to help solve this problem. These bacteria are relatively easy to cultivate, exhibit high resistance to abiotic conditions, and produce spores, which significantly enhance their durability and adaptability in the environment. Field studies provided results confirming the positive impact of *B. valezenzis* KT27 in reducing the effects of *C. beticola* infection. This was especially noted for the combination in which *B. valezenzis* KT27 was induced by adding an inactivated suspension of R*. cerealis* to the bacterial culture. This resulted in higher yields of sugar beet roots, compared to the control.

Many researchers confirm their exceptional metabolic potential, which translates into antagonistic properties against plant pathogens, the production of phytohormones, and their ability to solubilize phosphorus, potassium, silicon, zinc, and even assimilate atmospheric nitrogen. Considering all these beneficial characteristics of *Bacillus* bacteria, it is expected that they will increasingly become a key component of biofertilizers and biologically active products.

Although the conducted studies did not show very strong antagonistic properties against *R. cerealis* and *F. oxysporum*, the use of a preparation containing *B. velezensis* KT27 may still significantly support traditional methods for controlling diseases caused by these fungi.

**References**

1. Reda FM, Hassanein WA, Sherief EAH, Moabed S. Toxicological studies and field applications of a new *Bacillus thuringiensis* isolate (Bt1) and two chemical pesticides on *Spodoptera littoralis* (Boisd.) (*Lepidoptera*: *Noctuidae*). Egypt. J. Biol. Pest Control 2016; 26: 229–236.
2. Deguine JP, Aubertot JN, Flor RJ, Lescourret F, Wyckhuys KA, Ratnadass A. Integrated pest management: Good intentions, hard realities. A review. Agron. Sustain. Dev. 2021; 41: 1–35.
3. The European Council. 2019. New Green Deal, Communication from the Commission to the European Parliament, the European Council, the Council, The European Economic and Social Committee and the Committee Of The Regions The European Green Deal Com/2019/640 Final. https://eur-lex.europa.eu/legal-content/EN/TXT/?uri=COM%3A2019%3A640%3AFIN
4. Kurniawati A, Stankovics P, Hilmi YS, Toth G, Smol M, Toth Z. Understanding the future of bio-based fertilisers: The EU’s policy and implementation. Sustain. Chem. Clim. Act. 2023; 3: 100033. doi: 10.1016/j.scca.2023.10 0033.
5. Harman G, Khadka R, Doni F, Uphoff N. Benefits to plant health and productivity from enhancing plant microbial symbionts. Front. Plant Sci. 2021; 11: 610065. https://doi.org/10.3389/fpls.2020.610065.
6. Agricultural Biologicals Market Size, Share & Trends Analysis Report By Crop Type, By Product (Biopesticides, Biostimulants), By Application Method, By Region, And Segment Forecasts, 2024 – 2030. Report ID: GVR-4-68038-499-4.
7. Lahlali R, Ezrari S, Radouane N, Kenfaoui J, Esmaeel Q, El Hamss H, Belabess Z, Barka EA Biological Control of Plant Pathogens: A Global Perspective. Microorganisms 2022; 10: 596. https://doi.org/10.3390/microorganisms10030596.
8. Perez-Garcia O, Escalante FME, de-Bashan LE, Bashan Y. Heterotrophic cultures of microalgae: Metabolism and potential products. Water Res. 2011; 45: 11–36. https://doi.org/10.1016/j.watres.2010.08.037
9. Brader G, Compant S, Mitter B, Trognitz F, Sessitsch A. Metabolic potential of endophytic bacteria. Curr.Opin. Biotechnol. 2014; 27: 30–37. https://doi.org/10.1016/j.copbio.2013.09.012
10. Monzón-Atienza L, Bravo J, Torrecillas S, Montero D, Canales AFG-D, de la Banda IG, Galindo-Villegas J, Ramos-Vivas J, Acosta F. Isolation and Characterization of a *Bacillus velezensis* D-18 Strain, as a Potential Probiotic in European Seabass Aquaculture. Probiotics Antimicrob. Proteins 2021; 13: 1404–1412. doi: 10.1007/s12602-021-09782-8.
11. Silva LId, Pereira MC, Carvalho AMXd, Buttrós VH, Pasqual M, Dória J. Phosphorus-Solubilizing Microorganisms: A Key to Sustainable Agriculture. Agriculture. 2023; 13(2): 462. https://doi.org/10.3390/agriculture13020462
12. Cao Y, Xu Z, Ling N, Yuan Y, Yang X, Chen L, Shen B, Shen Q. Isolation and identification of lipopeptides produced by *B. subtilis* SQR 9 for suppressing *Fusarium* wilt of cucumber. Sci. Hort. 2012, 135, 32–39. https://doi.org/10.1016/j.scienta.2011.12.002.
13. Jiang C-H, Liao M-J, Wang H-K, Zheng M-Z, Xu J-J, Guo J-H. *Bacillus velezensis*, a potential and efficient biocontrol agent in control of pepper gray mold caused by *Botrytis cinerea*. Biol. Control. 2018; 126: 147–157. https://doi.org/10.1016/j.biocontrol.2018.07.017
14. Zeigler DR, Perkins JB. The genus *Bacillus*. *In* Practical Handbook of Microbiology; Goldman E, Green LH, Eds.; CRC Press: Boca Raton, FL, USA; 2018: pp. 309–326.
15. Shafi J, Tian H, Ji M. *Bacillus* species as versatile weapons for plant pathogens: A review. Biotechnol. Biotechnol. Equip. 2017; 31: 446–459. https://doi.org/10.1080/13102818.2017.1286950
16. Rabbee MF, Hwang B-S, Baek K-H. *Bacillus velezensis*: A Beneficial Biocontrol Agent or Facultative Phytopathogen for Sustainable Agriculture. Agronomy 2023; 13: 840. https://doi.org/10.3390/agronomy13030840
17. Collins DP, Jacobsen BJ. Optimizing a *Bacillus subtilis* isolate for biological control of sugar beet cercospora leaf spot. Biol. Control 2003; 26: 153–161. https://doi.org/10.1016/S1049-9644(02)00132-9
18. Rabbee MF, Ali MS, Choi J, Hwang BS, Jeong SC, Baek K. *Bacillus velezensis*: A valuable member of bioactive molecules within plant microbiomes. Molecules 2019; 24: 1046. https://doi.org/10.3390/molecules24061046.
19. Guo Y, Zhou J, Tang Y, Ma Q, Zhang J, Ji C, Zhao L. Characterization and genome analysis of a zearalenone-degrading *Bacillus velezensis* strain ANSB01E. Curr. Microbiol. 2020; 77: 273–278. doi: 10.1007/s00284-019-01811-8. Epub 2019 Nov 20.
20. Adeniji AALD, Babalola OO. *Bacillus velezensis*: Phylogeny, useful applications, and avenues for exploitation. Appl. Microbiol. Biotechnol. 2019; 103: 3669–3682. doi: 10.1007/s00253-019-09710-5
21. Zaid DS, Cai S, Hu C, Li Z, Li Y, Gralnick JA. Comparative genome analysis reveals phylogenetic identity of *Bacillus velezensis* HNA3 and genomic insights into its plant growth promotion and biocontrol effects. Microbiol. Spectr. 2022; 10: e02169-21. https://doi.org/10.1128/spectrum.02169-21.
22. Zaid D, Li W, Yang S, Li Y. Identification of bioactive compounds of *Bacillus velezensis* HNA3 that contribute to its dual effects as plant growth promoter and biocontrol against post-harvested fungi. Microbiol. Spectr. 2023; 11: e00519-23. https://doi.org/10.1128/spectrum.00519-23.
23. Lee SA, Kim SY, Sang MK, Song J, Weon HY. Complete genome sequence of *Bacillus velezensis* T20E-257, a plant growth-promoting bacterium, isolated from tomato (*Solanum lycopersicum* L.) root. Korean J. Microbiol. 2017; 53: 342–343. https://doi.org/10.7845/kjm.2017.7079
24. Xu Z, Xie J, Zhang H, Wang D, Shen Q, Zhang R. Enhanced control of plant wilt disease by a xylose-1 inducible degQ gene 2 engineered into *B. velezensis* strain SQR9XYQ. Phytopathology 2019; 109: 36-43. https://doi.org/10.1094/PHYTO-02-18-0048-R.
25. Bertrand S, Bohni N, Schnee S, Schumpp O, Gindro K, Wolfender J-L. Metabolite induction via microorganism co-culture: A potential way to enhance chemical diversity for drug discovery. Biotechnol. Adv. 2014; 32: 1180–1204. http://dx.doi.org/10.1016/j.biotechadv.2014.03.001.
26. Santos AM, Soares A, Luz J, Cordeiro C, Sousa Silva M, Dias T, Melo J, Cruz C, Carvalho L. Microbial Interactions as a Sustainable Tool for Enhancing PGPR Antagonism against Phytopathogenic Fungi. Sustainability 2024; 16: 2006. https://doi.org/10.3390/su16052006
27. Rangel LI, Spanner RE, Ebert MK, Pethybridge SJ, Stukenbrock EH, de Jonge R, Secor GA, Bolton MD. *Cercospora beticola*: The intoxicating lifestyle of the leaf spot pathogen of sugar beet. Mol. Plant Pathol. 2020; 21: 1020–1041. doi: 10.1111/mpp.12962.
28. Harveson R.M. Cercospora Leaf Spot of Sugar Beet. 2013. NebGuide. University of Nebraska-Lincoln Extension. (accessed 2024 October 22, https://extensionpubs.unl.edu/publication/g1753/2013/html/view).
29. Wolf PFJ, Verreet JA. Factors affecting the onset of Cercospora leaf spot epidemics in sugar beet and establishment of disease-monitoring thresholds. Phytopathology 2005; 95: 269–274. doi: 10.1094/PHYTO-95-0269.
30. Tan W, Li K, Liu D, Xing W. Cercospora leaf spot disease of sugar beet. Plant Signal Behav. 2023; 18: 2214765. https://doi.org/10.1080/15592324.2023.2214765.
31. Rosenzweig N, Hanson LE, Clark G, Franc GD, Stump WL, Jiang QW, Stewart J, Kirk WW. Use of PCR-RFLP Analysis to Monitor Fungicide Resistance in *Cercospora Beticola* Populations from Sugarbeet (*Beta vulgaris*) in Michigan, United States. Plant Dis. 2015; 99: 355–362. https://doi.org/10.1094/PDIS-03-14-0241-RE.
32. Rosenzweig N, Linda EH, Mambetova S, Jiang Q, Guza C, Stewart J, Trueman LC, Somohano P. Temporal population monitoring of fungicide sensitivity in *Cercospora beticola* from sugarbeet (*Beta vulgaris*) in the Upper Great Lakes. Can. J. Plant Pathol. 2020, 42, 469–479. https://doi.org/10.1080/07060661.2019.1705914.
33. Pikovskaya RI. Mobilization of Phosphorus in Soil Connection with the Vital Activity of Some Microbial Species. Microbiology, 1948; 17: 362-370
34. Rajawat MVS, Singh S, Tyagi SP, Saxena AK. A modified plate assay for rapid screening of potassium-solubilizing bacteria. Pedosphere 2016; 26: 768–773. https://doi.org/10.1016/S1002-0160(15)60080-7.
35. Bunt JS, Rovira AD. 1955. Microbiological Studies of Some Sub Antarctic Soils. J. Soil Sci. 1955; 6: 119-128.
36. European and Mediterranean Plant Protection Organization, 2002. Efficacy evaluation of plant protection products, PP 1/1(4) – Foliar diseases on sugarbeet, EPPO Standards.
37. European and Mediterranean Plant Protection Organization, 2014. Phytotoxicity assessment, PP 1/135(4), EPPO Standards.
38. European and Mediterranean Plant Protection Organization, 2012. Design and analysis of efficacy evaluation trials, PP1/152(4), EPPO Standards.
39. European and Mediterranean Plant Protection Organization, 2021. Conduct and reporting of efficacy evaluation trials, including good experimental practice, PP1/181(5), EPPO Standards.
40. European and Mediterranean Plant Protection Organization, 2012. Minimum effective dose, PP1/225(2), EPPO Standards.
41. Khan AR, Mustafa A, Hyder S, Valipour M, Rizvi ZF, Gondal AS, Yousuf Z, Iqbal R, Daraz U. *Bacillus* spp. as Bioagents: Uses and Application for Sustainable Agriculture. Biology 2022; 11: 1763. https://doi.org/10.3390/biology11121763.
42. Taghian, Shadia AM, Esh AZ Aly, Tohamy MRA. *Bacillus subtilis* as bioagent used to control cercospora sugar beet leaf spot disease. J. Agric. Res. 2008; 35(6): 1347-1360.
43. de Boer W, Hundscheid MP, Klein Gunnewiek PJ, de Ridder-Duine AS, Thion C, van Veen JA, van der Wal A, van Overbeek LS. Antifungal rhizosphere bacteria can increase as response to the presence of saprotrophic fungi. PLoS ONE 2015; 10: e0137988. doi:10.1371/journal.pone.0137988.
44. Leveau JHJ, Uroz S, De Boer W. The bacterial genus *Collimonas*: Mycophagy, weathering and other adaptive solutions to life in oligotrophic soil environments. Environ. Microbiol. 2010; 12: 281–292. https://doi.org/10.1111/j.1462-2920.2009.02010.x.
45. Hjort K, Lembke A, Speksnijder A, Smalla K, Jansson J. Community structure of actively growing bacterial populations in plant pathogen suppressive soil. Microb Ecol 2007; 53: 399–413. https://doi.org/10.1007/s00248-006-9120-2.
46. Falardeau J, Wise C, Novitsky L, Avis TJ. Ecological and Mechanistic Insights into the Direct and Indirect Antimicrobial Properties of Bacillus subtilis Lipopeptides on Plant Pathogens. J. Chem. Ecol. 2013; 39: 869–878. https://doi.org/10.1007/s10886-013-0319-7.
47. Farace G, Fernandez O, Jacquens L, Coutte F, Krier F, Jacques P, Clément C, Barka EA, Jacquard C, Dorey S. Cyclic lipopeptides from *Bacillus subtilis* activate distinct patterns of defence responses in grapevine. Mol. Plant Pathol. 2015; 16: 177–187. doi: 10.1111/mpp.12170.
48. Li Y, Héloir MC, Zhang X, Geissler M, Trouvelot S, Jacquens L, Henkel M, Su X, Fang X, Wang Q, Adrian M. Surfactin and fengycin contribute to the protection of a *Bacillus subtilis* strain against grape downy mildew by both direct effect and defence stimulation. Mol. Plant. Pathol. 2019; 20: 1037–1050. https://doi.org/10.1111/mpp.12809.
49. Valenzuela Ruiz V, Gándara-Ledezma A, Villarreal-Delgado MF, Villa-Rodríguez ED, Parra-Cota FI, Santoyo G, Gómez-Godínez LJ, Cira Chávez LA, de los Santos-Villalobos S. Regulation, Biosynthesis, and Extraction of Bacillus-Derived Lipopeptides and Its Implications in Biological Control of Phytopathogens. Stresses 2024; 4: 107-132. https://doi.org/10.3390/stresses4010007.
50. Klausmann P, Hennemann K, Hoffmann M, Treinen C, Aschern M, Lilge L, Heravi KM, Henkel M, Hausmann R. *Bacillus subtilis* High Cell Density Fermentation Using a Sporulation-Deficient Strain for the Production of Surfactin. Appl. Microbiol. Biotechnol. 2021; 105: 4141–4151. https://doi.org/10.1007/s00253-021-11330-x.
51. Lilge L, Ersig N, Hubel P, Aschern M, Pillai E, Klausmann P, Pfannstiel J, Henkel M, Morabbi Heravi K, Hausmann R. Surfactin shows relatively low antimicrobial activity against *Bacillus subtilis* and other bacterial model organisms in the absence of synergistic metabolites. Microorganisms 2022; 10: 779. https://doi.org/10.3390/microorganisms10040779.
52. Liu L, Jin X, Lu X, Guo L, Lu P, Yu H, Lv B. Mechanisms of Surfactin from *Bacillus subtilis* SF1 against *Fusarium foetens*: A Novel Pathogen Inducing Potato Wilt. J. Fungi 2023; 9: 367. https://doi.org/10.3390/jof9030367
53. Liu Y, Lu J, Sun J, Lu F, Bie X, Lu Z. Membrane disruption and DNA binding of *Fusarium graminearum* cell induced by C16-Fengycin A produced by *Bacillus amyloliquefaciens*. Food Control 2019; 102: 206–213. https://doi.org/10.1016/j.foodcont.2019.03.031.
54. El Housni Z, Ezrari S, Radouane N, Tahiri A, Ouijja A, Errafii K, Hijri M. Evaluating Rhizobacterial Antagonists for Controlling *Cercospora beticola* and Promoting Growth in *Beta vulgaris*. Microorganisms. 2024; 12(4): 668. doi: 10.3390/microorganisms12040668.
55. Arzanlou M, Mousavi S, Bakhshi M, Khakvar R, Bandehagh A. Inhibitory effects of antagonistic bacteria inhabiting the rhizosphere of the sugarbeet plants, on *Cercospora beticola* Sacc., the causal agent of Cercospora leaf spot disease on sugarbeet. J. Plant Prot. Res. 2016; 56: 6–14.
56. Esh AMH, El-Kholi MA, Taghian S. Antagonistic activities of *Bacillus amyloliquefaciens* from phyllosphere of sugar beet against *Cercospora beticola* Sacc. JPPP 2011; 2(1): 99–116. doi: 10.21608/jppp.2011.84660
57. Soliman SA, Abdelhameed RE, Metwally RA. In vivo and In vitro evaluation of the antifungal activity of the PGPR *Bacillus amyloliquefaciens* RaSh1 (MZ945930) against *Alternaria alternata* with growth promotion influences on *Capsicum annuum* L. plants. Microb. Cell Fact. 2023; 22: 70. https://doi.org/10.1186/s12934-023-02080-8.
58. Sehsah MD, El-Kot GA, El-Nogoumy BA, Alorabi M, El-Shehawi AM, Salama NH, El-Tahan AM. Efficacy of *Bacillus subtilis*, *Moringa oleifera* seeds extract and potassium bicarbonate on Cercospora leaf spot on sugar beet. Saudi J. Biol. Sci. 2022; 29: 2219–2229. doi: 10.1016/j.sjbs.2021.11.039.
