# Supplementary material for: Biocontrol of Cercospora leaf spot in sugar beet by a novel Bacillus velezensis KT27 strain: Enhanced antifungal activity and growth promotion in laboratory and field conditions
Source: PLoS One. 2025 May 30;20(5):e0323889. doi: 10.1371/journal.pone.0323889 (PMC12124551; doi:10.1371/journal.pone.0323889)
Supplement: Attachment — Submitted filename: Response to Reviewers.docx [file pone.0323889.s003.docx]

**Dear Reviewers,**

We would like to express our deepest gratitude for your thoughtful and constructive comments regarding our manuscript. Your insightful suggestions have been of great value to us, and we sincerely appreciate the time and effort you invested in reviewing our work.

We are confident that the revisions made in response to your remarks have considerably improved the scientific rigor, clarity, and overall quality of the manuscript. During the revision process, we also devoted careful attention to refining the language, as well as enhancing the technical and formal aspects of the manuscript.

To facilitate your review of the revised version, we have enabled the “Track Changes” feature to clearly indicate all substantial modifications. We have also included responses to your comments directly in the form of annotations. Nevertheless, we would kindly point out that the final, clean version of the manuscript best represents the full extent of the improvements made.

As part of the revision process, we have amended the manuscript title, which we believe now more accurately reflects the scope and essence of the study. Additionally, we have revised the titles of several subsections to improve structure and readability. Following your valuable suggestions, we have also updated the reference list and included photographs from the sugar beet field trials in which our bacterial biopreparation was applied.

We would also like to take this opportunity to clarify that the illustrative images provided reflect fungistatic activity observed under *in vitro* conditions. The well on the left side of the photograph demonstrates the activity of the bacterial biomass of *Bacillus velezensis* KT27, whereas the well on the right side corresponds to the post-culture fluid devoid of bacterial cells. We did not include images from the control treatment, as the application of sterile saline into wells on control plates did not result in any inhibition of fungal growth (i.e., no visible clearing zones were present). In our view, these would not contribute additional informative value.

We fully acknowledge that this is a broad and multifaceted topic, and that there are various possible approaches to its investigation. At the same time, we wish to emphasize that verifying laboratory results under field conditions—though often complex—is critical for confirming the efficacy of a biological preparation in practical agricultural settings. We stand by the reliability and validity of the results we have obtained.

While numerous studies are focused on identifying novel microbial strains with unique metabolic capacities for plant protection, we would like to highlight that the induction of fungistatic activity in microorganisms—such as the one we demonstrated—is still relatively underexplored. We sincerely hope that the publication of our findings will encourage further research others into this promising and important area.

We wholeheartedly agree with your recommendation to continue investigations along the lines outlined in the manuscript. Indeed, we are actively pursuing further research with the ultimate goal of formally registering our biopreparation, based on the *B. velezensis* KT27 strain, for protection against CLS, and eventually making it commercially available.

In our opinion, the subject of our research is well aligned with the increasingly urgent need to reduce the reliance on conventional chemical plant protection agents, as well as to mitigate the growing problem of pathogen resistance. These efforts, alongside a reduction in mineral fertilization, may substantially contribute to the improvement of environmental conditions impacted by modern agricultural practices.

Once again, we extend our sincere appreciation for your kind attention, careful review, and valuable input. We are truly grateful for the opportunity to improve our manuscript with your guidance.

With the highest respect and kind regards,

The Authors
